# Carbon Dots with an Emission in the Near Infrared Produced from Organic Dyes in Porous Silica Microsphere Templates

**DOI:** 10.3390/nano12030543

**Published:** 2022-02-05

**Authors:** Evgeniia A. Stepanidenko, Ivan D. Skurlov, Pavel D. Khavlyuk, Dmitry A. Onishchuk, Aleksandra V. Koroleva, Evgeniy V. Zhizhin, Irina A. Arefina, Dmitry A. Kurdyukov, Daniil A. Eurov, Valery G. Golubev, Alexander V. Baranov, Anatoly V. Fedorov, Elena V. Ushakova, Andrey L. Rogach

**Affiliations:** 1 Center of Information Optical Technologies, ITMO University, Kronverksky Pr. 49, 197101 Saint Petersburg, Russia; stepanidenko.e@mail.ru (E.A.S.); ivan.skurlov.23@gmail.com (I.D.S.); onishchuk.dmitry@gmail.com (D.A.O.); irina-arefina97@mail.ru (I.A.A.); a_v_baranov@yahoo.com (A.V.B.); a_v_fedorov@inbox.ru (A.V.F.); 2 Chair of Physical Chemistry, TU Dresden, Zellescher Weg 19, 01069 Dresden, Germany; pavel.khavlyuk@tu-dresden.de; 3 Centre for Physical Methods of Surface Investigation, Saint Petersburg State University, Universitetskaya emb. 7-9, 199034 Saint Petersburg, Russia; dalika@inbox.ru (A.V.K.); evgeny_liquid@mail.ru (E.V.Z.); 4 Laboratory of Amorphous Semiconductors, Ioffe Institute, 26 Politekhnicheskaya Str., 194021 Saint Petersburg, Russia; kurd.gvg@mail.ioffe.ru (D.A.K.); edan@mail.ru (D.A.E.); golubev.gvg@mail.ioffe.ru (V.G.G.); 5 Centre for Functional Photonics (CFP), Department of Materials Science and Engineering, City University of Hong Kong, Hong Kong 999077, China; andrey.rogach@cityu.edu.hk; 6 Shenzhen Research Institute, City University of Hong Kong, Shenzhen 518057, China

**Keywords:** carbon dots, template synthesis, silica microspheres, near infrared emission, organic dyes

## Abstract

Carbon dots (CDs) with an emission in the near infrared spectral region are attractive due to their promising applications in bio-related areas, while their fabrication still remains a challenging task. Herein, we developed a template-assisted method using porous silica microspheres for the formation of CDs with optical transitions in the near infrared. Two organic dyes, Rhodamine 6G and IR1061 with emission in the yellow and near infrared spectral regions, respectively, were used as precursors for CDs. Correlation of morphology and chemical composition with optical properties of obtained CDs revealed the origin of their emission, which is related to the CDs’ core optical transitions and dye-derivatives within CDs. By varying annealing temperature, different kinds of optical centers as derivatives of organic dyes are formed in the microsphere’s pores. The template-assisted method allows us to synthesize CDs with an emission peaked at 1085 nm and photoluminescence quantum yield of 0.2%, which is the highest value reported so far for CDs emitting at wavelengths longer than 1050 nm.

## 1. Introduction

Alongside well-known light-emitting materials such as organic dyes, rare-earth elements and semiconductor quantum dots, luminescent nanomaterials based on carbon, such as carbon dots (CDs), are gaining much interest [1,2]. This is because of their advantageous optical and physicochemical properties including high photo- and chemical stability, strong absorption in the UV and visible spectral regions, efficient photoluminescence (PL) and low cytotoxicity [3,4,5]. Besides, CDs can be prepared from inexpensive renewable natural sources [6]. Due to these useful characteristics, CDs and composite materials based thereon have been suggested for use in biomedicine [7,8,9], optoelectronics [10,11], sensing [6,12], as luminescent inks [13], as initiators for polymerization [14], and even in agriculture to improve plant growth [15].

Among different synthetic techniques used for CD fabrication, bottom-up methods are most popular, where organic molecular precursors undergo several stages of transformation during the synthesis: condensation, polymerization, aromatization, carbonization, and passivation [16]. The obtained CDs often demonstrate bright PL and do not require subsequent surface functionalization; their PL quantum yield (QY) can reach more than 90% [17]. The most common synthetic routes are hydro-/solvothermal treatment and microwave-assisted pyrolysis [18,19]. However, the main obstacle of these methods is the necessity of post-synthetic purification of synthesized products from unreacted molecules and the eventually formed organic fluorophores. Another limitation of solvothermal and microwave-assisted methods is the reaction temperature range dictated by the boiling point of a solvent. The template-assisted method, on the other hand, allows those disadvantages to be overcome together with provision of pressure control; it also decreases the influence of solvents involved in chemical reaction and formation of the products. Another advantage of the template-assisted method is the possibility of shape and size control by architecture of template, which may result in CDs with higher monodispersity without additional purification procedures [20]. Among different templates suitable for CD synthesis, reversed micelles [21], zeolites [22,23], metal-organic frameworks [24,25], and silica [26] or calcium carbonate microspheres [27] have been reported. Thus, CDs can be formed both at the surface of the template as was shown for CDs synthesized on silica colloid spheres [28], and inside the template as was shown for CDs carbonized within emulsion micelles [29]. The latter approach gives more control of CDs’ morphology. For instance, the degree of CDs’ carbonization depends on their localization in CaCO3 microparticles: in the external shell layer CDs with a morphology similar to graphene oxide were formed, while in the inner cavities less-ordered nanoparticles were formed [27]. By using the template-assisted method, CDs with optical transitions in the visible spectral range can be formed. In reference [26] blue-emissive CDs formed from citric acid in mesoporous silica spheres with excitation dependent as well as up-converted emission were reported. In our previous work [30], it was shown that using microporous silica microspheres, CDs can be synthesized using organic dyes as precursors. However, the origin of optical transitions, and the dependence of the types of optical centers formed on the synthetic parameters still need to be established for the template-assisted method. Yet another advantage of the template synthesis is the ability to directly produce functional composite materials based on CDs. For instance, composites of CDs in zeolites demonstrated delayed PL at ambient conditions due to stabilization of the triplet states of CDs by zeolite frameworks [31].

One of the current challenges is the synthesis of CDs with PL in the near infrared (NIR) range, which are desirable for bioimaging [32,33,34,35,36]. The PL QY of the long-wavelength emission of CDs sharply decreases when moving from the red to NIR spectral range: it was reported to be over 64% for the PL peak at ≈600 nm [37] but decreased to 10% at 700–800 nm [38,39,40], and further lowered to 1% at 900–1000 nm [41]. Several examples of CDs with optical transitions in deep red/NIR spectral region are provided in Table S1 (Supporting Information). Most of these CDs were prepared by “classical” methods such as solvothermal synthesis; thus, the adoption and/or expansion of them for NIR emissive CDs is a promising way for further development of the CDs’ application areas.

In this work, a template-assisted method for the formation of red and NIR-emissive CDs by annealing of organic dye precursors, Rhodamine 6G and IR1061, in the pores of mesoporous silica microspheres is developed. From comprehensive investigation of morphology and optical properties, the origin of optical transitions for CDs was derived. The formed CDs are homogeneous nanoparticles consisting of heteroatom-doped carbon aromatic network and dye-derivatives. This morphology results in the appearance of optical transitions in the visible and NIR spectral range associated with carbon network and dye-derivatives, respectively. For NIR emissive CDs, the dependence of the optical centers formation on annealing temperature has been established for the first time. The template-assisted method introduced here allows us to synthesize CDs with an emission peaked at 1085 nm and photoluminescence quantum yield of 0.2% which is the highest value reported so far for CDs emitting at the wavelengths longer than 1050 nm. Thus, it opens a way to produce CDs with near infrared emission, which are in high demand for bioimaging and sensors.

## 2. Materials and Methods

### 2.1. Materials

Rhodamine 6G (Rh) and IR1061 dyes were purchased from Sigma-Aldrich (Darmstadt, Germany). Ethanol (>96%), methanol (≥99.8%), chloroform (≥99.8%), acetone (≥99.9%), 25% aqueous ammonia solution (≥99.9%) and 40% hydrofluoric acid (≥99.9%) were purchased from “Vekton” (Saint Petersburg, Russia). All reagents were used as received. Ultrapure water (Milli-Q) was used throughout the experiments.

As templates, microspheres of mesoporous silica with a diameter of 490 ± 90 nm, a pore volume of ~50% and an average pore diameter of 3.0 ± 0.2 nm were used. These particles were synthesized according to previous work [42].

### 2.2. Carbon Dot (CD) Synthesis

**Scheme 1:**
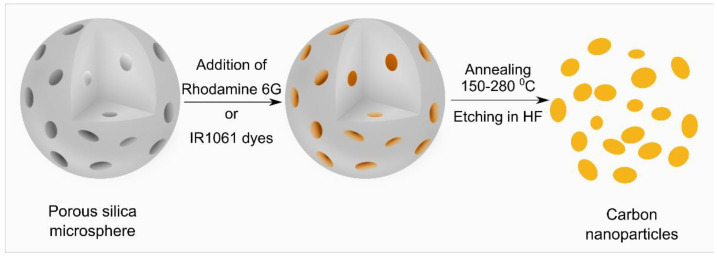
Fabrication of carbon dots (CDs) from organic dyes by template-assisted method in silica microspheres.

The synthesis of CDs was carried out in the pores of silica microspheres, based on the procedure adopted from reference [30] where CDs were synthesized from Rh. The preliminary results showed that the template-assisted method developed in our previous works resulted in the synthesis of CDs with reproducible optical properties (Table S2). Firstly, silica spheres were impregnated with a dye solution (200 mM of Rh in methanol, or 2 mM of IR1061 in chloroform). Then, the samples were dried and heated to a required temperature with a 10 deg/min step in an oven with a programmable controller. The heat treatment was carried out under normal conditions in air for 2 h at different temperatures in the range of 150–280 °C, namely at 280 °C for Rh, and at 150, 200 or 250 °C for IR1061. Finally, silica microsphere templates were removed by etching in HF solution. The CDs obtained were thoroughly washed to eliminate the residual HF. Schematics of the fabrication of CDs is given in Scheme 1, while chemical formula of the organic dye precursors used, and photographs of the resulting CD solutions, are given in Figure S1 (Supporting Information). The CDs obtained were designated as “Rh-CDs” for CDs based on Rh; and “NIR-CDs-150”, “NIR-CDs-200” and “NIR-CDs-250” for CDs based on IR1061 dye and annealed at 150, 200 or 250 °C, respectively. Reference samples were prepared by heating up IR1061 dye in chloroform in an oven at 150 and 200 °C for 1 h; they were designated as “IR1061-150” and “IR1061-200”, respectively. For further optical measurements, Rh-CDs were dissolved in an 25% aqueous solution of ammonia, while NIR-CDs were dissolved in acetone.

### 2.3. Experimental Setup

Atomic force microscopy (AFM) measurements were carried out using a Solver PRO-M microscope (NT-MDT) in the semi-contact mode. For AFM measurements, solutions of CDs were spin-coated onto the mica substrates at 2000 rpm for 30 s. Raman spectra were recorded using a micro-Raman spectrometer InVia (Renishaw) with a 514.5 nm line of the Ar^+^ laser in the backscattering geometry, using a 50× Leica objective (NA = 0.78). The laser power at the sample’s surface was fixed at the level excluding any sample heating. Fourier-transform infrared (FTIR) spectra were recorded on a Tenzor II infrared spectrophotometer (Bruker, Billerica, MA, USA) in an attenuated total reflection mode. X-ray photoelectron spectroscopy (XPS) was carried out using an ESCALAB 250Xi photoelectron spectrometer (Thermo Fisher Scientific, Waltham, MA, USA) with AlKα radiation (photon energy of 1486.6 eV) in the constant pass energy mode at 100 eV for the survey spectrum, and at 50 eV for the element core level spectra using an XPS spot size of 650 μm. The peaks were fitted with Voigt functions. Absorption spectra were recorded on a spectrophotometer UV-3600 (Shimadzu, Kyoto, Japan). PL spectra in the UV-Vis range were collected on a FP-8200 spectrofluorometer (Jasco, Tokyo, Japan) and Cary Eclipse (Agilent, Santa Clara, CA, USA). Near-infrared PL was measured using a custom build spectrofluorometer [43], using the LDH-P-C-980 laser (PicoQuant, Berlin, Germany) with a wavelength of 980 nm and output power 3.9 mW as an excitation source. Resulting spectra were corrected using the blackbody radiation source lamp (SLS201 Thorlabs, Newton, NJ, USA) [44]. PL QYs were estimated relative to standards: Rhodamine 6G for visible and spherical PbS quantum dots in tetrachlorethylene with an average size of 3.9 ± 0.2 nm and PL QY = 20%, synthesized by the hot-injection method according to [45], for IR spectral regions. Time-resolved PL measurements were performed on a confocal microscope MicroTime 100 (PicoQuant, Berlin, Germany) equipped with a 3× objective (NA = 0.1) and a 405 nm pulsed diode laser. PL decay curves were fitted by a biexponential function: $I\left(t\right)=I_{0}+A_{1}e^{-t/\tau_{1}}+A_{2}e^{-t/\tau_{2}}$. The average PL lifetime has been calculated as $\langle \tau \rangle =\sum A_{i}\tau_{i}^{2}/\sum A_{i}\tau_{i}$.

## 3. Results and Discussion

### 3.1. Origin of the Rh-CDs Emission

**Figure 1:**
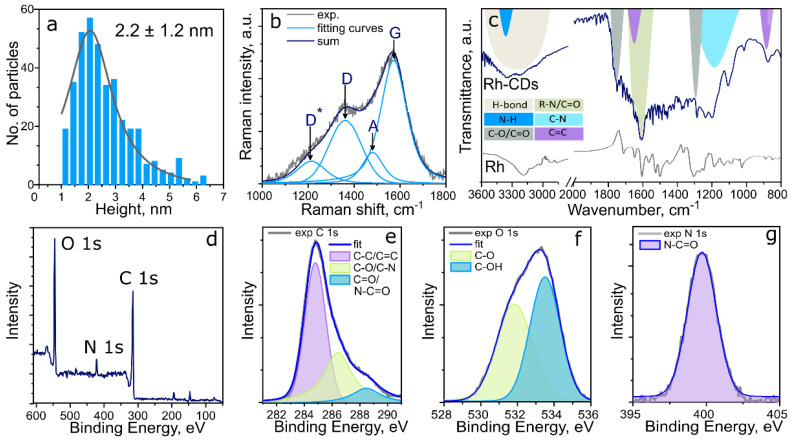
(**a**) Atomic force microscopy (AFM) height distribution of Rhodamine (Rh)-CDs. (**b**) Raman spectrum of Rh-CDs (exp., grey) and its deconvolution (sum, dark blue) into four peaks (fitting curves, light blue). (**c**) Fourier transform infrared (FTIR) spectra of Rh-CDs (dark blue) and Rh (grey); different types of molecular groups are shown by different color areas. (**d**) Full X-ray photoelectron spectroscopy (XPS) survey spectrum of Rh-CDs, and high-resolution XPS spectra of Rh-CDs: C1S (**e**), O1S (**f**), and N1S (**g**), with deconvolution of bands to specific peaks attributed to different chemical groups shown by different colors and explained in the legends.

To study the morphology and chemical composition of the CDs obtained, we used a combination of spectroscopy and microscopy methods, including AFM, XPS, FTIR and Raman spectroscopy. The AFM method was used to study the height of CDs. According to AFM images of Rh-CDs, the height of Rh-CDs varies in the range of 1–7 nm, with an average value of 2.2 ± 1.2 nm (Figure 1a). Lateral sizes of Rh-CDs are much larger than their height (Figure S2), which can be explained by the formation of graphene layers from the xanthene groups of Rh in a stacked manner. Raman spectroscopy was carried out to investigate the inner morphology of the CDs. The Raman spectrum of Rh-CDs (Figure 1b) consists of 4 bands: D* (1211 cm^−1^), D (1359 cm^−1^), A (1480 cm^−1^) and G (1571 cm^−1^) which are typical for carbon polymorphs and CDs [46]. The most intense D and G bands correspond to breathing mode near the graphene layer edge and stretching mode of the sp^2^ carbon network, respectively. The D* band is attributed to stretching vibrations of sp^2^–sp^3^ bonds in disordered graphitic lattice as well as vibrations of trans-polyacetylene chains [47,48]. The A band is typical for sp^3^-hybridized amorphous carbon [49]. From the G band peak position (1570 cm^−1^) and the ratio of the intensities (I) of the D and G bands I(D)/I(G) ≈ 1, according to the amorphization trajectory for carbon polymorphs [46] it can be inferred that the structure of synthesized Rh-CDs is intermediate between nanocrystalline graphite and amorphous carbon with the sp^3^ content less than 10%. Taking into account that these CDs should contain some oxygen, the Raman spectra can be compared with that of graphene oxide and reduced graphene oxide. In reference [50] it was shown that the ratio of the intensities (I) of the TPA and G bands I(TPA)/I(G) indicate the amount of oxygen in the sample. From the comparison of the published data [50] and the value I(TPA)/I(G) ≈ 0.2 for Rh-CDs, the amount of oxygen in CDs is estimated to be approximately 20%.

In order to study the chemical composition of the CDs, as well as to compare them with the precursor used, the FTIR spectra were registered. A comparison of FTIR spectra of Rh-CDs and the Rh precursor shows that alongside with peaks inherited from the precursor, several additional bands appeared (Figure 1c). The intensity of the broad absorption band at 3000–3400 cm^−1^ increases together with a decrease of the bands at 2800–3000 cm^−1^. This observation is attributed to the formation of O–H and N–H bonds, which happens parallel to a decrease of the amount of C–H aliphatic bonds present in Rh. Absorption in the range of 1750–1100 cm^−1^ increases significantly, with an emergence of broad peaks at {1750, 1280}, {1650, 1610}, and {1250–1070} cm^−1^ which are typical for C=O/C–O stretching mode, carboxylic acid derivatives (including amides), and stretching vibrations of C–N groups, respectively. It should be noted that the increased absorption at 1650 cm^−1^ can also be caused by formation of an aromatic C=C network. Moreover, peaks attributed to the C–N groups within the aromatic carbon network prevail as compared to Rh.

To further study the chemical composition of Rh-CDs, XPS measurements were taken. From the full survey XPS spectrum (Figure 1d), the Rh-CDs consist of 65% carbon, 6% nitrogen, and 25% oxygen. The residual fluorine amount is approximately 4% (Figure S3). It is worth mentioning that the estimated amount of oxygen from Raman spectra is consistent with that from XPS analysis. Figure 1e–g show high-resolution XPS spectra for C 1S, O 1S, and N 1S bands and their decomposition into peaks corresponding to various bonds as presented in Figure’s legends. The C1S band is deconvoluted to peaks at 284.8, 286.4, and 288.5 eV (Figure 1e), which are attributed to C–C/C=C carbon network, C–OH/C–O–C/C–N bonds, and R–C=O bonds, respectively. The O 1S band is deconvoluted to two peaks at 531.8 and 533.5 eV (Figure 1f), which correspond to C–O–C/O–C=O and C(aromatic)–OH bonds, respectively. The N 1S peak at 399.7 eV (Figure 1g) is attributed to the amide (N–C=O) bonds. These findings agree well with those from the FTIR analysis. Thus, Rh-CDs mostly consist of the aromatic C=C network with carboxylic acid derivatives (O–C=O/N–C=O) and have –OH/–NH groups at the surface.

**Figure 2:**
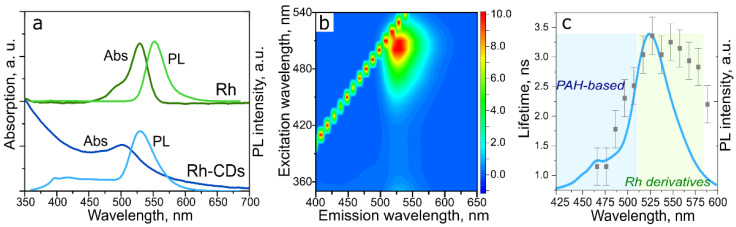
Optical properties of Rh-CDs in 25%-aq. ammonia solution. (**a**) Absorption (Abs) and photoluminescence (PL) spectra (excited at 350 nm) of Rh in ethanol (upper panel) and Rh-CDs (lower panel). (**b**) the PL excitation-emission (PLE-PL) map of Rh-CDs. (**c**) Average PL lifetimes (grey squares) at different wavelengths, shown together with the PL spectrum excited at 405 nm (blue line); error bars represent the instrument error.

Optical properties of Rh-CDs are illustrated in Figure 2. Compared to Rh absorption (Figure 2a, upper panel), Rh-CDs demonstrate an increased absorption in the short-wavelength region (less than 450 nm) (Figure 2a, lower panel), which is attributed to the O-doped aromatic carbon network formed. The main absorption peak which is inherited from the Rh absorption band at 532 nm is broadened and blue shifted to 500 nm. The intense PL band inherited from the Rh precursor follows the trends for the absorption spectrum: it is blue shifted to 532 nm as compared to 554 nm for Rh, with an increase of its full width at half maximum (FWHM) from 29 to 39 nm. The appearance of a weak PL signal at shorter-wavelength region can be attributed to the formation of optical centers originating from small polycyclic aromatic hydrocarbons (PAH) and their derivatives [51]. In Figure 2b, the PL excitation-emission (PLE-PL) map demonstrates that the emissive optical centers corresponding to Rh derivatives can be excited via energy transfer from the PAH-based moieties with intense absorption in shorter-wavelength region [52]. To confirm this assumption, transient PL measurements were carried out. As shown in Figure 2c, the average PL lifetime depends on the PL wavelength, and thus two regions can be distinguished where average PL lifetime linearly depends on PL wavelength or saturates at approximately 3.2 ns. According to the literature [53], the PL lifetime for Rh in methanol at the optimal concentration equals to 3.7 ns; with an increase of Rh concentration the PL lifetime can decrease down to 2.5 ns as a result of the excimer formation. Since these PL lifetime values of Rh are similar to that observed in the 2nd region in Figure 2c, we can assume that, indeed, the longer-wavelength emission originates from the Rh derivatives. The values of PL lifetime in the first region varies from approximately 1.1 to 2.5 ns, which is approximately one order of magnitude less than those for PAHs (from 12 to 20 ns) [54,55,56]. Considering the evidence of energy transfer from blue-emissive centers (PAHs) to Rh derivatives within Rh-CDs as seen in the PLE-PL map (Figure 2b), it can be inferred that this energy transfer is a dynamic process; as a result, both PL intensity and the lifetime of Rh-CDs are decreased in a nonlinear manner. Thus, in the Rh-CDs synthesized by the template method here, optical centers attributed to PAH-based moieties and dye derivatives are formed, with an efficient energy transfer occurring to longer-wavelength emissive counterparts.

### 3.2. Origin of the Near Infrared (NIR)-CD Emission

**Figure 3:**
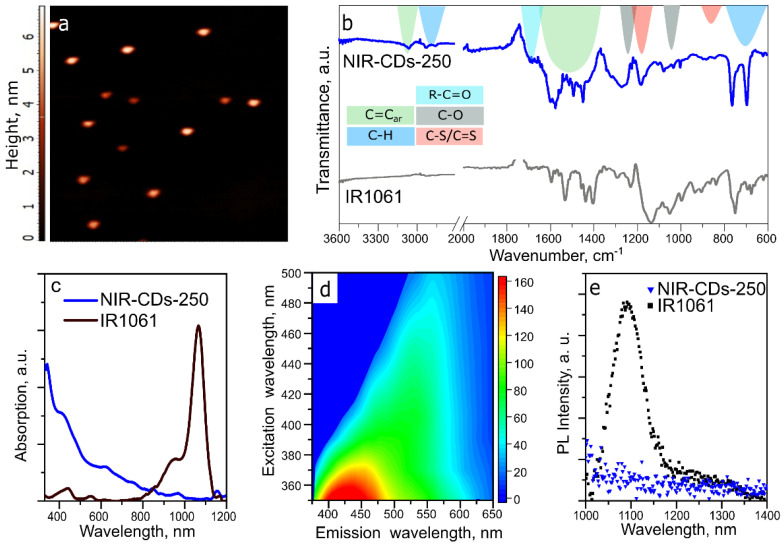
Morphology and optical properties of near infrared (NIR)-CDs-250. (**a**) AFM image (image size: 1.7 × 2 μm). (**b**) FTIR and (**c**) absorption spectra, compared with those of the IR1061 precursor. (**d**) PLE-PL map. (**e**) Emission in the NIR spectral region from NIR-CD-250 in acetone, compared with emission of IR1061 in chloroform.

To shift the optical transitions to deep-red and NIR spectral region, an IR dye (IR1061) with a PL band at approximately 1085 nm was chosen to accomplish the template-assisted synthesis of NIR-CDs (see Scheme 1). Firstly, NIR-CDs were synthesized using a similar procedure as for Rh-CDs, where the temperature was set at 250 °C. The CDs formed, which were designated as NIR-CDs-250, were found to be rather homogeneous particles with a height of 4–6 nm as determined by AFM (Figure 3a). FTIR analysis revealed that the change of the precursor (IR1061) spectrum follows the trend described above for Rh-CDs, namely an increase of absorption in the region of 1700–1200 cm^−1^ corresponding to the stretching vibrations of aromatic carbon network and C=O/C–O groups (Figure 3b). Thus, it could be expected that the longer-wavelength optical transition should be inherited by these CDs from the IR dye. However, the spectral analysis of NIR-CDs-250 showed that NIR bands are absent in absorption and PL spectra (Figure 3c–e). Instead of the absorption bands at 1064 and 945 nm which are typical for IR1061, an increase of absorption in the visible spectral region is observed for NIR-CDs-250 with peaks at 410 and 625 nm. The PL-PLE map (Figure 3d) demonstrates that blue-emissive CDs are formed with the most intense PL signal observed at 430 nm when excited at 350 nm, with PL peak position dependent on the excitation wavelength typical for CDs [57]. The NIR PL signal is absent in this type of CDs as shown in Figure 3e. Thus, at 250 °C all optical centers responsible for NIR PL are decomposed, and instead blue-emissive moieties are formed. 

### 3.3. Temperature-Dependent Formation of NIR-CDs

It was previously shown for solvothermal synthesis that the reaction temperature affects both structural and optical properties of formed CDs [57]: at temperatures below 180 °C alongside CDs lots of molecular fluorophores are formed, while upon an increase of temperature up to 300 °C the type of emissive centers are changed significantly [58]. Thus, the temperature dependence of morphology and optical properties of NIR-CDs synthesized by the template-assisted method here was examined further. To track the changes in the resulting nanoparticles and to establish the ongoing stages of synthesis, including the formation of precursor aggregates, no purification procedures were used. NIR-CDs-150 and NIR-CDs-200 were synthesized at temperatures 150 and 200 °C, respectively; their properties were compared to NIR-CDs-250 and IR1061 dye heated up to the same temperatures.

**Figure 4:**
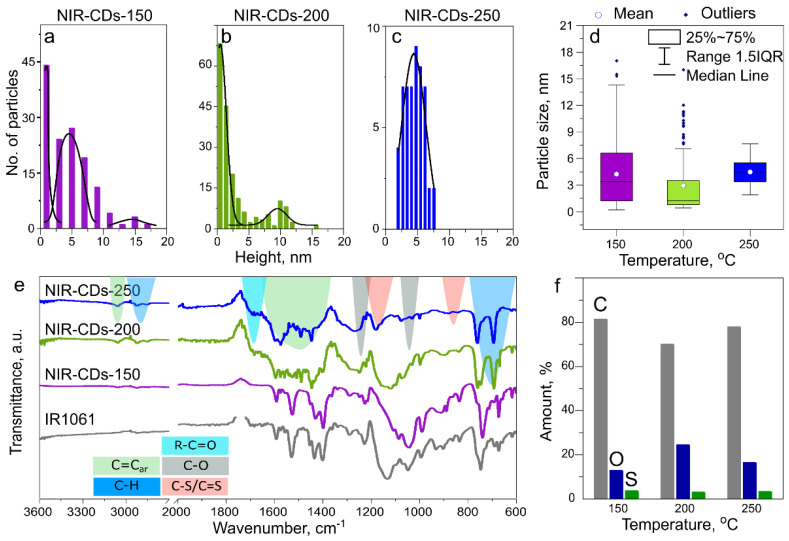
Morphology of NIR-CDs dependent on the temperature of synthesis: height distribution obtained from the analysis of AFM images for NIR-CDs-150 (**a**), NIR-CDs-200 (**b**), and NIR-CDs-250 (**c**); (**d**) statistics of the NIR-CDs heights depending on annealing temperature (IQR—interquartile range); (**e**) FTIR spectra; (**f**) chemical composition determined from XPS measurements.

The analysis of AFM images (Figure S4) showed that the shape and size distribution of NIR-CDs formed depends significantly on annealing temperature. From the height distribution diagrams (Figure 4a–c), it can be seen that several fractions are formed. For NIR-CD-150, there are three types of particle: (i) moieties smaller than 1 nm, which probably corresponds to the molecular residues of the precursor; (ii) particles with a height of 1–8 nm with an average value of 5 nm; (iii) 10–18 nm particles which can be attributed to aggregates. With an increase of annealing temperature to 200 °C, the redistribution of fractions is observed: most of the particles possess height up to 4 nm, with some fraction of 8–12 nm particles/aggregates. The most homogeneous sample is NIR-CDs-250 with an average height of 4.4 ± 1.1 nm. The statistical analysis of particles’ height (Figure 4d) showed that upon increasing annealing temperature the height distribution decreases, with a disappearance of some fractions of particles shown as outlier data points in Figure 4d. Only for NIR-CDs-250 the mean value coincides with the median value, while for NIR-CDs-150 and NIR-CDs-200 the median value is less than the mean one, pointing on a broader height distribution in those two cases. The particle height distribution decreases with an increase of temperature, but the mean value is the lowest for NIR-CDs-200.

Raman spectra of all NIR-CDs contain all the bands typical for CDs, including D*, D, A, and G as shown in Figure S5a–c. It should be noted that the Raman signal for NIR-CDs-150 and NIR-CDs-200 varies from point to point as shown in Figure S5d,e, which is yet another hint on the inhomogeneity of their structure. According to the amorphization trajectory for carbon polymorphs [46], it can be inferred that the structure of synthesized NIR-CDs is intermediate between the nanocrystalline graphite and the amorphous carbon with sp^3^ content at around 10%; and a smaller sp^3^ content is observed for NIR-CDs-200 (Figure S5f). Furthermore, FTIR spectra showed that NIR-CDs-150 possess almost the same structure as the IR1061 precursor, with decreased stretching vibrations of C–S bonds (peak at approximately 1200 cm^−1^) which points to decomposition of the precursor to smaller moieties (Figure 4e). When the temperature is higher than 200 °C, peaks typical for carbon nanoparticles emerge: the absorption increases in the range 1750–1500 cm^−1^ associated with stretching vibrations of the aromatic carbon domain and carboxylic derivatives shown by cyan and green areas in Figure 4e. The full XPS survey spectrum (Figure S6) showed that all NIR-CDs consist of C, O, and S elements, with ratios provided in Figure 4f. Oxygen atoms are absent in the precursor IR1061 molecule (Figure S1), but they are introduced during an annealing procedure. The S content of 2–3% is independent of annealing temperature, while both the C and O amounts vary. The C content varies from 81% to 70% and 77% for NIR-CDs formed at 150, 200, and 250 °C, respectively. The O content demonstrates an opposite dependence, varying from 13% to 25% and 17% for NIR-CDs formed at 150, 200, and 250 °C, respectively. It should be noted that the residual fluorine amount is below 3% (Figure S7). High-resolution XPS spectra revealed the formation of different bonds within NIR-CDs (Figure S8). For all the samples, the C 1s band consists of two peaks centered at 284.8 and 286.3 eV, which are attributed to C–C/C–H and C–OH/C–O–C, respectively. It should be noted that the C–S band may be hidden within the 284.5–285.0 eV energy region [59,60]. The S 2p band consists of spin-orbit coupled 2p 3/2 and 1/2 of C–S bonds at 163.9 and 165.1 eV [61], and of a peak at 168.2 eV which can be associated with the S–O bond in –C–SO_x_ –(x = 3) [62]. The O 1S band consists of a single peak centered at 532.6, 532.6, and 532.4 eV for NIR-CDs-150, NIR-CDs-200, and NIR-CDs-250, respectively. Hence, the process of the precursor’s oxidation during annealing within the template depends on temperature and results in the oxidation of sulfur, and formation of C–O–C aliphatic bonds at 150–200 °C, which transform to ketone groups (C=O/O–C=O) at 250 °C. These findings agree well with the FTIR spectra, especially for the change in the oxygen bonding.

From the analysis of morphology, it can be inferred that: at 150 °C precursor aggregates/cross-linked precursors are formed with a broad size distribution; at 200 °C alongside with those aggregates, carbonized particles with a high degree of oxidation are formed; and at 250 °C, homogeneous O-doped carbon nanoparticles are formed. These findings are similar to those observed for CD formation during microwave-assisted pyrolysis at 240 °C [63], where the particles undergo several structural configurations, starting from (i) aggregation of organic molecules, (ii) core and shell formation, (iii) shell collapse, and (iv) formation of aromatic groups within the core. It is worth mentioning that in contrast to IR1061 and other NIR emissive dyes [64] which are soluble only in non-polar solvents, NIR-CDs synthesized here are soluble in polar solvents such as acetone and water, due to their carboxylic derivative groups. This suggests that NIR emissive CDs developed in this work are promising for bioimaging and sensing in cells and tissues.

**Figure 5:**
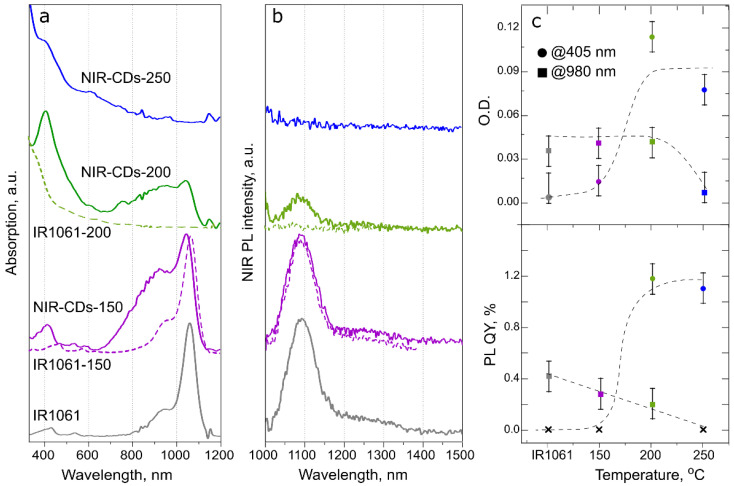
Optical properties of NIR-CDs and the related reference samples produced at different temperatures. (**a**) Absorption, and (**b**) PL spectra (excited at 980 nm) of the organic dye IR1061 (grey), NIR-CD-150 (magenta), NIR-CD-200 (green), and NIR-CD-250 (blue), compared with reference samples IR1061-150 (dashed magenta line) and IR1061-200 (dashed green line). (**c**) Optical density (upper panel) and PL quantum yield (QY, lower panel) of the NIR-CDs versus annealing temperature, excited at 405 nm (circles) and 980 nm (squares). Black crosses demonstrate the absence of any PL signal; error bars represent the instrument/estimation error.

The annealing temperature also affects the optical properties of NIR-CDs. As shown in Figure 5a, with the temperature increase the NIR absorption band broadens and shifts from 1065 nm to 1050 nm for NIR-CDs-150 and NIR-CDs-200, together with an increase of optical density of the shoulder at 940–950 nm. It should be noted that the reference sample of IR dye heated at 150 °C demonstrates almost the same absorption spectrum as the original dye as shown in Figure 5a by dashed magenta line, while the increase of heating temperature up to 200 °C results in a complete dye decomposition as shown by dashed green line. For NIR-CDs-200 and NIR-CDs-250, an increase of optical density in the 300–700 nm region with the most intense band at 405 nm is observed, indicating formation of an O-doped aromatic carbon network. The shape of PL spectra of NIR-CDs-150 and NIR-CDs-200 almost coincide with that of IR1061. Even the reference sample IR1061-150 demonstrates the PL band similar to the original dye (dashed magenta line in Figure 5b). However, the IR1061-200 sample has no NIR PL signal as shown in Figure 5b by the dashed green line. Alongside with NIR emissive centers based on IR1061 dye, an excitation-dependent emission in the visible spectral range is observed for NIR-CDs-200 and NIR-CDs-250 (Figure 3d and Figure S9), which is typical for CDs. It is worth noting that the reference sample IR1061-200 also demonstrates PL in the visible spectral region, with an excitation dependent peak position (Figure S10); however, its PL intensity is much lower compared to that of NIR-CDs-200.

Figure 5c presents the trends in optical density and PL QYs (measured under excitation at 405 and 980 nm associated with the O-doped aromatic carbon network and IR-dye derivatives, respectively), upon annealing temperature used to produce the samples studied. It shows that only at 250 °C optical centers associated with NIR absorption are decomposed, while IR1061 dye is decomposed at lower temperatures. Moreover, the formation of carbonized particles takes place at temperatures higher than 150 °C. The increase of the optical density in the visible spectral region for NIR-CDs-200 and NIR-CDs-250 agrees well with their morphology analysis provided above, besides larger optical density at 405 nm of NIR-CDs-200 as compared to NIR-CDs-250 which could be associated with increased oxidation degree of the carbon network. NIR PL QY decreases with annealing temperature starting from 0.42% (IR1061) to almost 0.28% (NIR-CDs-150) and 0.20% (NIR-CDs-200), while PL QY in the visible spectral region for NIR-CDs-200 and NIR-CDs-250 are independent of annealing temperature. Hence, the template-assisted method used in this work allows us to obtain CDs from IR-dye molecules with preservation of their emissive centers alongside the formation of aromatic carbon network with optical transitions in the visible spectral range.

## 4. Conclusions

In summary, the template-assisted method used to synthesize CDs from organic dyes leads to the formation of nanoparticles whose charged surface groups allow for their easy redispersion in polar solvents. For CDs based on Rhodamine 6G, two types of optical center are formed which originated from the carbon aromatic network (PAH) and dye derivatives, including their aggregates. The formation of these optical centers depends on the annealing temperature, as shown on CDs produced from the IR1061 dye. Increase of the temperature results in the formation and further growth of a carbon aromatic network with optical transitions in the visible spectral range, while dye-based optical centers are decomposed resulting in the decrease of optical density and emission intensity in the NIR spectral range. Thus, we were able to produce NIR emissive CDs with a PL band at 1085 nm, and PLQY of 0.2%, which is the highest value published so far for CDs emitting at the wavelengths longer than 1050 nm. We conclude that the template-assisted method applied here is a useful tool for the synthesis of carbon nanoparticles with controlled properties via use of different organic dyes and annealing temperatures.

## Supplementary Materials

The following are available online at https://www.mdpi.com/article/10.3390/nano12030543/s1, Table S1. Comparison of optical properties of NIR-emissive CDs; Table S2. Properties of CDs synthesized by template-assisted method using silica microspheres; Figure S1. Photographs of the CD samples prepared from Rhodamine 6G (left, Rh-CDs)) and IR1061 (right, NIRT-CDs), taken under day light illumination (“Vis”) and under UV-lamp (“UV”); Figure S2. AFM image of Rh-CDs; Figure S3. XPS survey spectra of fluorine in Rh-CDs. Fluorine atomic content is approximately 4%; Figure S4. AFM images of (a) NIR-CDs-150, (b) NIR-CDs-200, and (c) NIR-CDs-250; Figure S5. Raman spectra of NIR-CDs excited at 633 nm: (a,d) NIR-CDs-150, (b,e) NIR-CDs-200, and (c) NIR-CDs-250 (c). On panels (a–c), experimental data (exp.) are shown in black, deconvoluted peaks corresponding to D*, D, A, and G bands (fit. curves)—in green, and the overall curves (sum)—in red. Raman spectra shown in panels (d,e) illustrate inhomogeneity of NIR-CDs-150 and NIR-CDs-200, respectively. (f) G band peak positions and I(D)/I(G) values for NIR-CDs samples (NIR-CD-150, NIR-CD-200, NIR-CD-250) versus annealing temperature, and approximate values the of sp^3^ content from amorphization trajectories for carbon polymorphs; Figure S6. XPS survey spectra of (a) NIR-CDs-150, (b) NIR-CDs-200, and (c) NIR-CDs-250. Atomic ratios of constituting elements derived from these spectra are provided as insets; Figure S7. XPS survey spectra of fluorine in NIR-CDs-150, NIR-CDs-200, and NIR-CDs-250. Fluorine content is estimated as 2.9, 3.0, and 2.8% for NIR-CDs-150, NIR-CDs-200, and NIR-CDs-250, respectively; Figure S8. High resolution XPS spectra of C1S (a,d,g), O1S (b,e,h), and S2p (c,f,i) of NIR-CDs-150 (a–c), NIR-CDs-200 (d–f), NIR-CDs-250 (g–i); Figure S9. (a) PLE and (b) PL spectra of NIR-CDs-200 in acetone. Emission and excitation wavelengths are listed in the legends; Figure S10. (a) PLE and (b) PL spectra of IR1061-200 in chloroform. Emission and excitation wavelengths are listed in the legends. References [20,30,37,38,39,40,41,65,66,67,68] are cited in the Supplementary Materials.
