# Supplementary material for: Carbon Dots with an Emission in the Near Infrared Produced from Organic Dyes in Porous Silica Microsphere Templates
Source: Nanomaterials (Basel). 2022 Feb 5;12(3):543. doi: 10.3390/nano12030543 (PMC8838831; doi:10.3390/nano12030543)
Supplement: Supplementary file 1 [file nanomaterials-12-00543-s001.zip › nanomaterials-1566120-supplementary-final.pdf]

SUPPORTING MATERIAL

Carbon Dots with an Emission in the Near Infrared Produced from Organic Dyes in Porous Silica Microsphere Templates

Evgeniia A. Stepanidenko [1], Ivan D. Skurlov [1], Pavel D. Khavlyuk [2], Dmitry A. Onishchuk [1], Aleksandra V. Koroleva [3], Evgeniy V. Zhizhin [3], Irina A. Arefina [1], Dmitry A. Kurdyukov [4], Daniil A. Eurov [4], Valery G. Golubev [4], Alexander V. Baranov [1], Anatoly V. Fedorov [1], Elena V. Ushakova [1,*] and Andrey L. Rogach [5,6]

[1] Center of Information Optical Technologies, ITMO University, Kronverksky pr. 49, 197101 Saint Petersburg, Russia; stepanidenko.e@mail.ru (E.A.S.); ivan.skurlov.23@gmail.com (I.D.S.); onishchuk.dmitry@gmail.com (D.A.O.); irina-arefina97@mail.ru (I.A.A.); a_v_baranov@yahoo.com (A.V.B.); a_v_fedorov@inbox.ru (A.V.F.)

[2] Chair of Physical Chemistry, TU Dresden, Zellescher Weg 19, 01069 Dresden, Germany; pavel.khavlyuk@tu-dresden.de

[3] Centre for Physical Methods of Surface Investigation, Saint Petersburg State University, Universitetskaya emb. 7–9, 199034 Saint Petersburg, Russia; dalika@inbox.ru (A.V.K.); evgeny_liquid@mail.ru (E.V.Z.)

[4] Laboratory of Amorphous Semiconductors, Ioffe Institute, 26 Politekhnicheskaya Str., 194021 Saint Petersburg, Russia; kurd.gvg@mail.ioffe.ru (D.A.K.); edan@mail.ru (D.A.E.); golubev.gvg@mail.ioffe.ru (V.G.G.)

[5] Centre for Functional Photonics (CFP), Department of Materials Science and Engineering, City University of Hong Kong, Hong Kong 999077, China; andrey.rogach@cityu.edu.hk

[6] Shenzhen Research Institute, City University of Hong Kong, Shenzhen 518057, China

* Correspondence: elena.ushakova@itmo.ru

Table S1. Comparison of optical properties of NIR-emissive CDs

Precursors	Synthesis method (solvent), temperature, and reaction time	Size, nm	Absorption peak(s)/range [nm]	PL maximum[nm]	PL QY @ λ_{ex}, %	Ref.
O-phenylenediamine, dopamine	Hydrothermal, 200 °C, 6 h	5.6	223, 280, 615	648 and 685 (λ_{ex} = 560 nm)	34	[1]
O-phenylenediamine, urea	Hydrothermal, 210 °C, 8 h	2.6	280, 537, 570	600, 650	20	[2]
P-phenylenediamine, $NiCl_2$	Hydrothermal, 160 °C, 6 h	2.9	510	605	65	[3]
Citric acid, urea	Solvothermal ([1]DMF), 160 °C, 6 h; MW-assisted exfoliation in DMF, 70 °C, 70 min	4 (diameter), 0.4–2 (height)	619, 720	770 (λ_{ex} = 732 nm)	11@732 nm	[4]
Citric acid, urea, ammonium fluoride	Solvothermal (DMF), 180 °C, 4 h	2.6	556, 624, 715, 847	658 and 777 (fs-laser, λ_{ex} > 700 nm)	10@710 nm	[5]
Citric acid, 1,4,5,8-tetramino-anthraquinon	Hydrothermal, 180 °C, 2 h	2.45 (diameter), 0.94 (height)	230, 280, 650	700	7–11	[6]
PEG800 and CyOH	Solvothermal (ethanol), 160 °C, 2 h	2.9	783	820	6	[7]
Watermelon juice	Solvothermal, 190 °C, 3 h	6	400–800	925 (λ_{ex} = 808 nm)	0.4	[8]
IR1061	Template-assisted, 200 °C, 2 h	4	350–1050	1080	0.2	This work

[1]*DMF—dimethylformamide*

Table S2. Properties of CDs synthesized by template-assisted method using silica microspheres

Sample precursor	Size, nm	Absorption band, nm	Photoluminescence band, nm	Reference
APTES	3.3	250–450	425	[9,10]
APTES	3.6	250–450	425	[11]
Rhodamine 6G	3.1 *	475–550	510–560	[9]
Rhodamine 6G	2.2	475–550	510–560	This work

* *pore size of silica microspheres used as a template.*

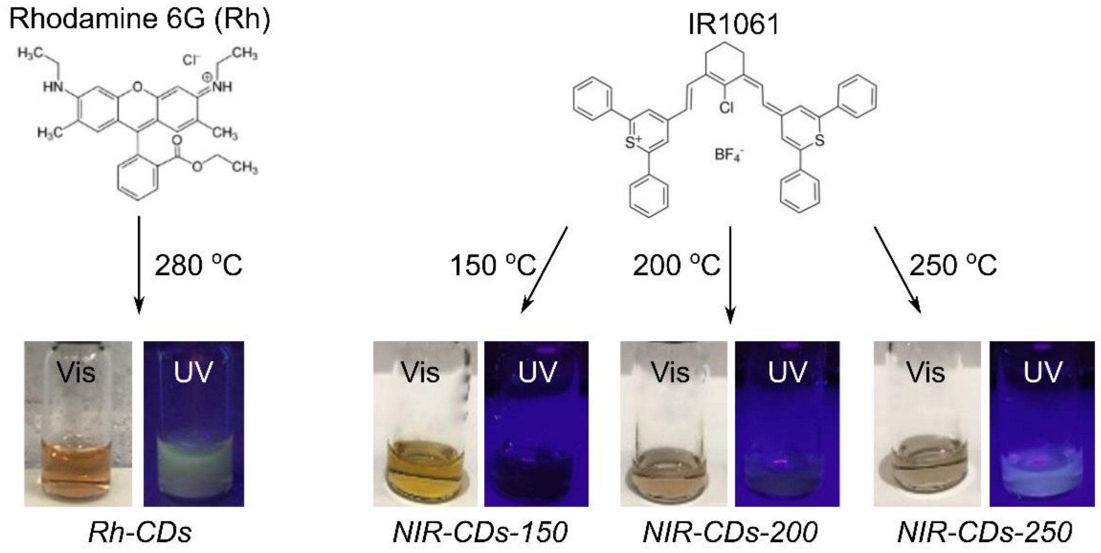

Figure S1. Photographs of the CD samples prepared from Rhodamine 6G (left, Rh-CDs)) and IR1061 (right, NIRT-CDs), taken under day light illumination ("Vis") and under UV-lamp ("UV").

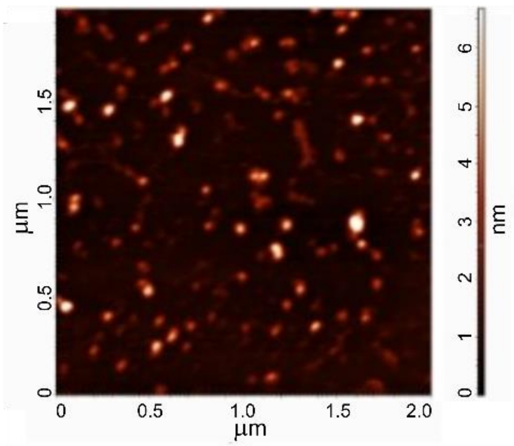

Figure S2. AFM image of Rh-CDs.

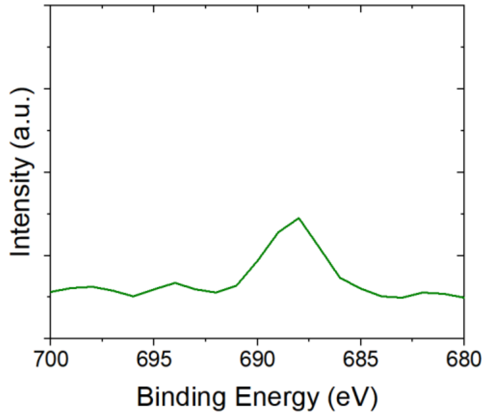

Figure S3. XPS survey spectra of fluorine in Rh-CDs. Fluorine atomic content is approx. 4%.

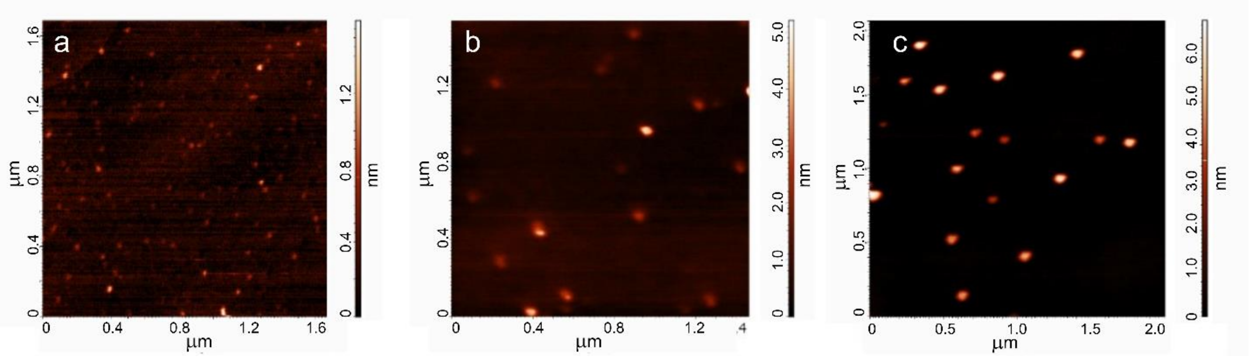

Figure S4. AFM images of (**a**) NIR-CDs-150, (**b**) NIR-CDs-200, and (**c**) NIR-CDs-250.

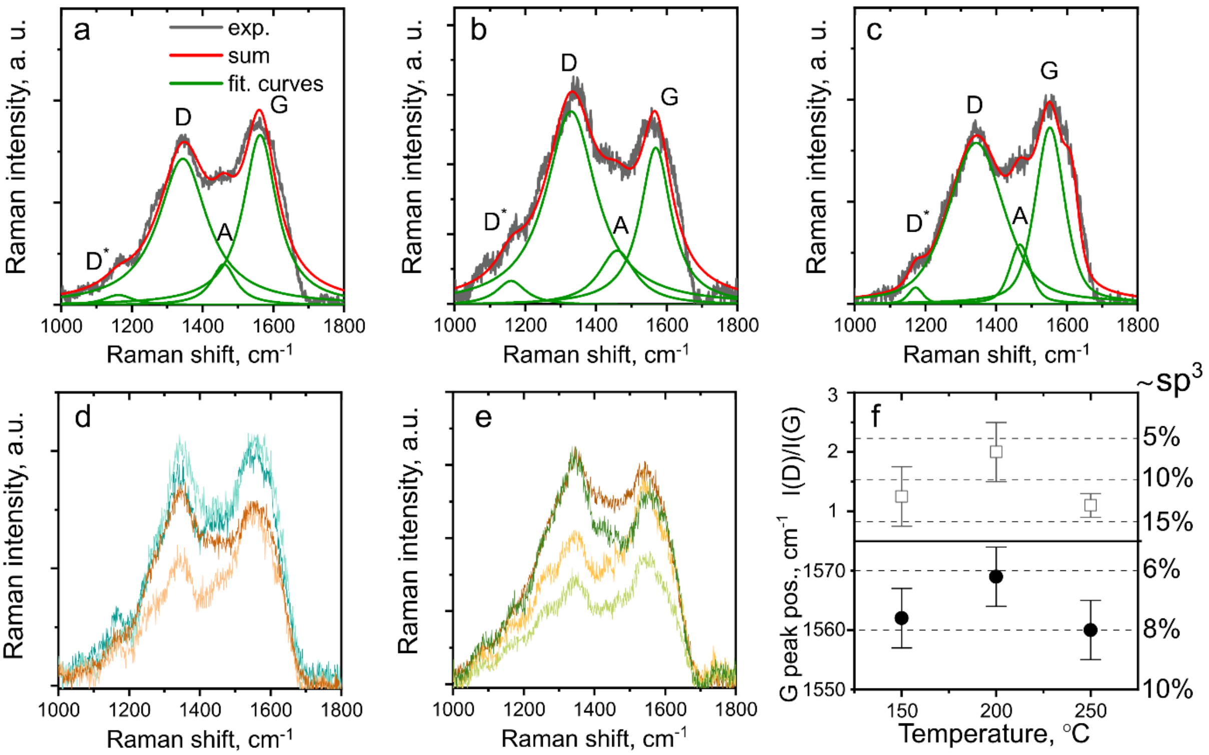

Figure S5. Raman spectra of NIR-CDs excited at 633 nm: (**a,d**) NIR-CDs-150, (**b,e**) NIR-CDs-200, and (**c**) NIR-CDs-250 (**c**). On panels (**a–c**), experimental data (exp.) are shown in black, deconvoluted peaks corresponding to D*, D, A, and G bands (fit. curves)—in green, and the overall curves (sum)—in red. Raman spectra shown in panels (**d**) and (**e**) illustrate inhomogeneity of NIR-CDs-150 and NIR-CDs-200, respectively. (**f**) G band peak positions and I(D)/I(G) values for NIR-CDs samples (NIR-CD-150, NIR-CD-200, NIR-CD-250) versus annealing temperature, and approximate values the of sp^3 content from amorphization trajectories for carbon polymorphs.

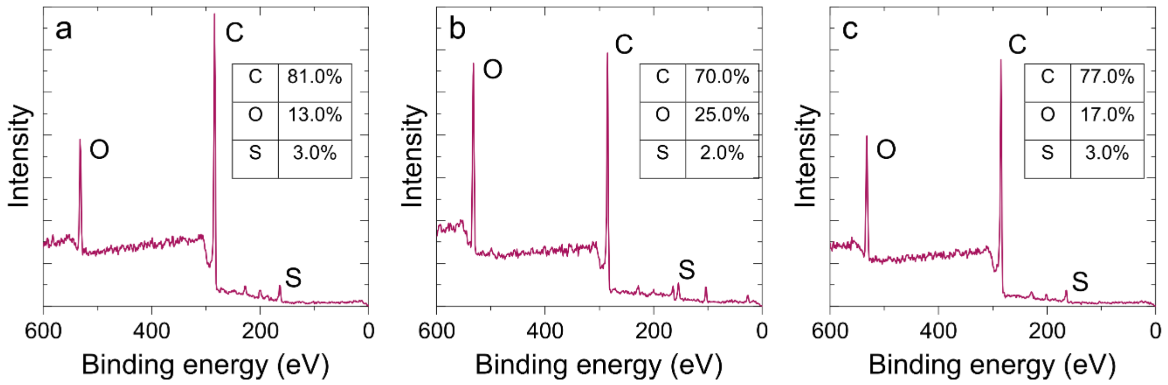

Figure S6. XPS survey spectra of (**a**) NIR-CDs-150, (**b**) NIR-CDs-200, and (**c**) NIR-CDs-250. Atomic ratios of constituting elements derived from these spectra are provided as insets.

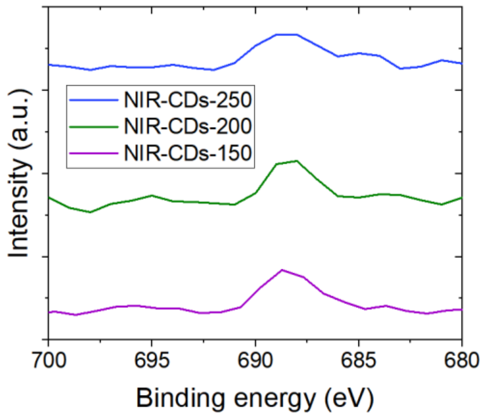

Figure S7. XPS survey spectra of fluorine in NIR-CDs-150, NIR-CDs-200, and NIR-CDs-250. Fluorine content is estimated as 2.9, 3.0, and 2.8% for NIR-CDs-150, NIR-CDs-200, and NIR-CDs-250, respectively.

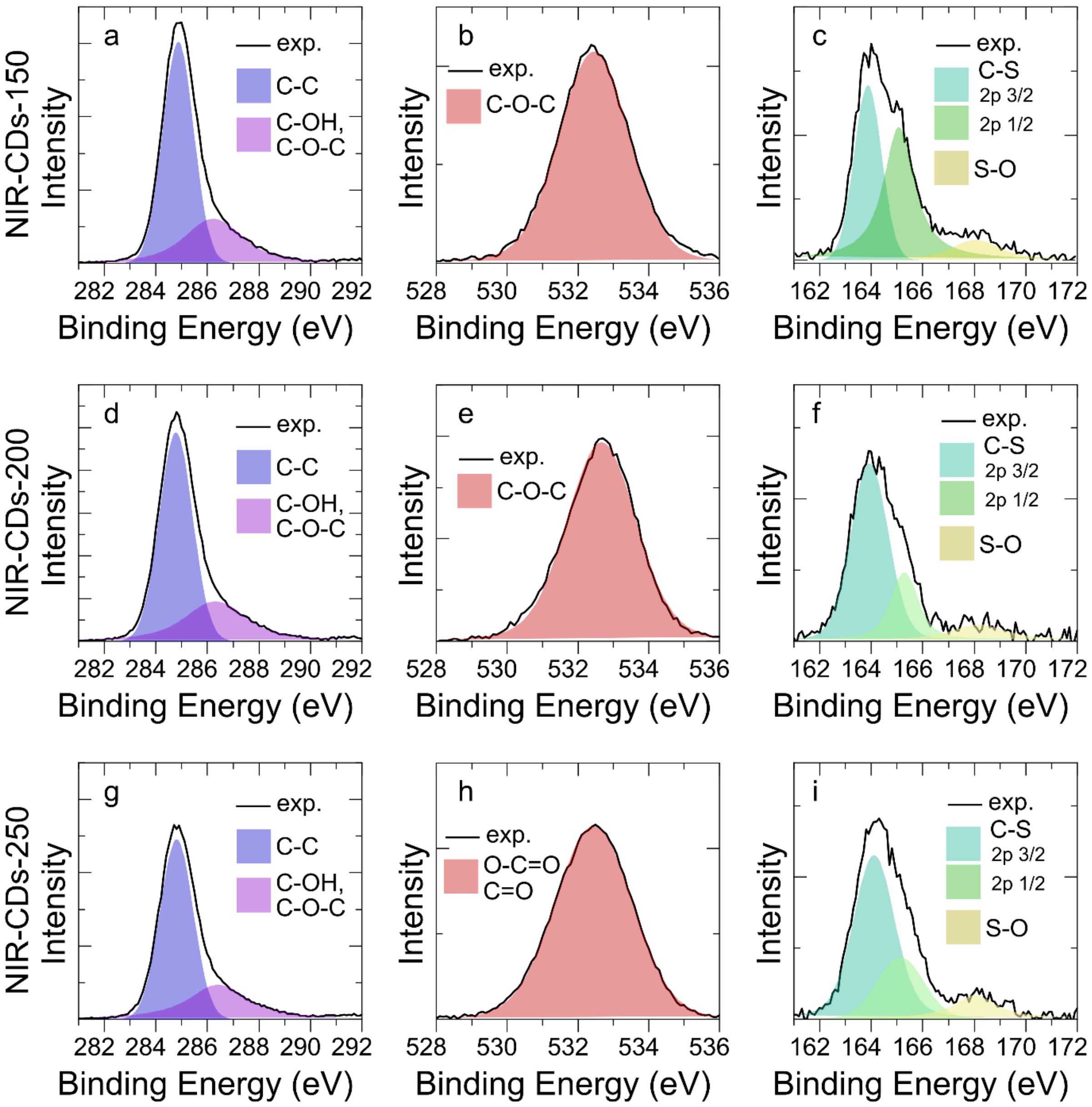

Figure S8. High resolution XPS spectra of C1S (**a,d,g**), O1S (**b,e,h**), and S2p (**c,f,i**) of NIR-CDs-150 (**a–c**), NIR-CDs-200 (**d–f**), NIR-CDs-250 (**g–i**).

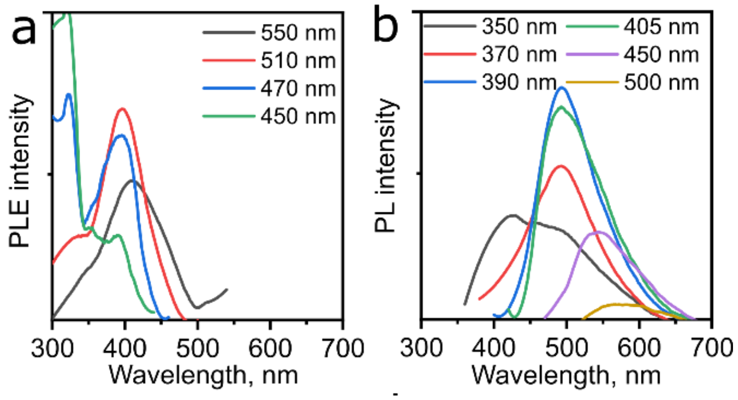

Figure S9. (**a**) PLE and (**b**) PL spectra of NIR-CDs-200 in acetone. Emission and excitation wavelengths are listed in the legends.

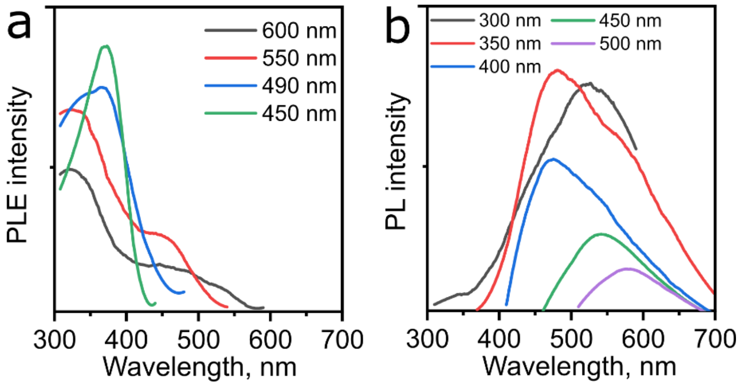

Figure S10. (**a**) PLE and (**b**) PL spectra of IR1061-200 in chloroform. Emission and excitation wavelengths are listed in the legends.

References

1. Wang, B.; Li, J.; Tang, Z.; Yang, B.; Lu, S. Near-Infrared Emissive Carbon Dots with 33.96% Emission in Aqueous Solution for Cellular Sensing and Light-Emitting Diodes. *Science Bulletin* **2019**, *64*, 1285–1292. https://doi.org/10.1016/J.SCIB.2019.07.021.
2. Zhu, Z.; Liu, C.; Song, X.M.; Mao, Q.; Ma, T. Carbon Dots as an Indicator of Acid-Base Titration and a Fluorescent Probe for Endoplasm Reticulum Imaging. *ACS Applied Bio Materials* **2021**, *4*, 3623–3629. https://doi.org/10.1021/acsabm.1c00121.
3. Hua, X.-W.; Bao, Y.-W.; Zeng, J.; Wu, F.-G. Nucleolus-Targeted Red Emissive Carbon Dots with Polarity-Sensitive and Excitation-Independent Fluorescence Emission: High-Resolution Cell Imaging and in Vivo Tracking. *ACS Applied Materials & Interfaces* **2019**, *11*, 32647–32658. https://doi.org/10.1021/ACSAMI.9B09590.
4. Li, D.; Liang, C.; Ushakova, E. v; Sun, M.; Huang, X.; Zhang, X.; Jing, P.; Jo Yoo, S.; Kim, J.-G.; Liu, E.; et al. Thermally Activated Upconversion Near-Infrared Photoluminescence from Carbon Dots Synthesized via Microwave Assisted Exfoliation. *Small* **2019**, *15*, 1905050. https://doi.org/10.1002/SMLL.201905050.
5. Jiang, L.; Ding, H.; Xu, M.; Hu, X.; Li, S.; Zhang, M.; Zhang, Q.; Wang, Q.; Lu, S.; Tian, Y.; et al. UV–Vis–NIR Full-Range Responsive Carbon Dots with Large Multiphoton Absorption Cross Sections and Deep-Red Fluorescence at Nucleoli and In Vivo. *Small* **2020**, *16*, 2000680. https://doi.org/10.1002/smll.202000680.
6. Li, S.; Su, W.; Wu, H.; Yuan, T.; Yuan, C.; Liu, J.; Deng, G.; Gao, X.; Chen, Z.; Bao, Y.; et al. Targeted Tumour Theranostics in Mice via Carbon Quantum Dots Structurally Mimicking Large Amino Acids. *Nature Biomedical Engineering 2020 4:7* **2020**, *4*, 704–716. https://doi.org/10.1038/s41551-020-0540-y.

7 Zheng, M.; Li, Y.; Liu, S.; Wang, W.; Xie, Z.; Jing, X. One-Pot to Synthesize Multifunctional Carbon Dots for Near Infrared Fluorescence Imaging and Photothermal Cancer Therapy. *ACS Applied Materials and Interfaces* **2016**, *8*, 23533–23541. https://doi.org/10.1021/acsami.6b07453.

8 Li, Y.; Bai, G.; Zeng, S.; Hao, J. Theranostic Carbon Dots with Innovative NIR-II Emission for in Vivo Renal-Excreted Optical Imaging and Photothermal Therapy. *ACS Applied Materials and Interfaces* **2019**, *11*, 4737–4744. https://doi.org/10.1021/acsami.8b14877.

9 Eurov, D.A.; Kurdyukov, D.A.; Medvedev, A. v; Golubev, V.G. Synthesis of Monodisperse Carbon Nanodots with Variable Photoluminescence Spectrum Using Polyaromatic Precursors. *Technical Physics Letters* **2019**, *45*, 940–942. https://doi.org/10.1134/S1063785019090232.

10 Kurdyukov, D.A.; Eurov, D.A.; Stovpiaga, E.Y.; Kirilenko, D.A.; Konyakhin, S. v.; Shvidchenko, A. v.; Golubev, V.G. Template Synthesis of Monodisperse Carbon Nanodots. *Physics of the Solid State* **2016**, *58*, 2545–2549. https://doi.org/10.1134/S1063783416120167.

11 Rabchinskii, M.; Mironov, L.; Sgibnev, Y.; Kolesnikov, I.; Kurdyukov, D.; Eurov, D.; Kirilenko, D.; Shvidchenko, A.; Stolyarova, D.; Smirnov, D.; et al. Fluorescence Enhancement of Monodisperse Carbon Nanodots Treated with Aqueous Ammonia and Hydrogen Peroxide. *Nanotechnology* **2019**, *30*, 475601. https://doi.org/10.1088/1361-6528/ab3cdf.
